# Mothers’ Experiences of Childbirth and Perspectives on Korean Medicine-Based Postpartum Care in Korea: A Qualitative Study

**DOI:** 10.3390/ijerph19095332

**Published:** 2022-04-27

**Authors:** Do-Eun Lee, Hyo-Weon Suh, Han-Song Park, Inae Youn, Minjung Park, Joohee Seo

**Affiliations:** 1 Department of Neuropsychiatry, College of Korean Medicine, Won Kwang University, Iksan 54538, Korea; lde401@wku.ac.kr; 2 Department of Korean Medicine, College of Korean Medicine, Kyung Hee University, Seoul 02453, Korea; hyoweonsuh@gmail.com; 3 Department of Clinical Korean Medicine, Graduate School, Kyung Hee University, Seoul 02447, Korea; kikihansong@nmc.or.kr (H.-S.P.); eknowkey@nmc.or.kr (I.Y.); 4 National Agency for Development of Innovative Technologies in Korean Medicine, National Institute of Korean Medicine Development, 12F Namsan Square Building, 173 Toegyero, Jung-gu, Seoul 04553, Korea; mj.park@nikom.or.kr or; 5 Department of Korean Neuropsychiatry, National Medical Center, 245 Euljiro, Jung-gu, Seoul 04564, Korea

**Keywords:** postpartum care, postnatal depression, Korean medicine, thematic analysis, qualitative research

## Abstract

This study aimed to record the experiences of childbirth and postpartum care of postpartum women and gain an in-depth understanding of their experiences of Korean medicine-based postpartum healthcare. The investigator conducted a 60–90-min interview with the 8 participants (mean age 34 years), and the comments were analyzed using the thematic analysis method. The two major themes emerging from the participants’ comments were: “experience and awareness of childbirth and postpartum care” and “experiences of the Korean medicine-based postpartum program”. The first theme was analyzed in four primary categories: (1) experiences of breakdown of the body and mind; (2) impossibility of postpartum care without help; (3) experiences of relentless effort for recovery; and (4) experiences of body and mind recovery. The second theme was analyzed in four primary categories: (1) participation with vague expectations; (2) experiences of the effects of managing postpartum symptoms; (3) the need for a comprehensive Korean medicine management for postpartum women; and (4) suggested improvements for the Korean medicine-based postpartum program. Mothers recognized the importance of Korean medicine treatment during the postpartum period for the management of Sanhupung symptoms and postpartum care and reported the benefits of body warming, Sanhupung prevention, pain reduction, and sense of psychological stability.

## 1. Introduction

Postpartum women experience various changes as they adapt to the role of being a mother, including physical discomfort following childbirth, changes in endocrine function, and psychological problems [1]. Without proper care during the postpartum period, there may be a risk of complications for both the mother and the child [2,3]. Furthermore, postpartum depression is considered of great significance in international postpartum care clinical guidelines [4]. This is because the prevalence of postpartum depression is approximately 10–20%, and without proper treatment, it can lead to chronic depression [5]. In severe cases, it may lead to infanticide or suicide [6], and, thus, it is crucial to implement aggressive intervention strategies for postpartum depression starting from the primary care setting to create a healthy childbirth and parenting environment.

In Korean medicine (KM), childbirth is viewed as a big event that exhausts vital substances, such as vital energy (Qi) and blood (Xue), due to bleeding and overexertion during childbirth, depleted fluids and humors, and easy infiltration by pathogens [7]. In this regard, there is the concept of “Sanhupung” as a cultural disease related to postpartum care [8]. Sanhupung is a KM term that synthesizes various postpartum symptoms caused by improper management of the postpartum period after childbirth or miscarriage [8]. It can be classified into three types of symptoms as follows: pain-related symptoms, systemic symptoms, and psychological symptoms. Psychological symptoms in Sanhupung refer to various symptoms caused by postpartum stress, and are not the same as diagnostic postpartum depression [8].

KM interventions to treat Sanhupung include acupuncture, electroacupuncture, herbal medicine, moxibustion, cupping, and chuna. Electroacupuncture is an improvement of traditional acupuncture as a treatment that stimulates acupuncture points with electricity instead of manual manipulation [9]. Moxibustion is a treatment that prevents and treats diseases by heating specific areas of the body surface by regulating the function of meridians by burning certain types of substances [10]. Cupping is considered physical therapy; by sucking in the skin, it increases the local circulation of blood and lymph and relieves painful muscle tension [11]. Chuna is a Korean spinal manipulation technique consisting of stimulation of the meridians, correction of the displacement of the bone joint structure, and prescription of exercise according to the differentiation of symptoms and functional and anatomical evaluation [12]. Many studies have been conducted on KM-based interventions applied during the postpartum period. According to a satisfaction survey of Korean mothers, 85.24% of women reported greater satisfaction with postpartum care that included KM treatment than with other types of postpartum care that they received following previous pregnancies [13], whereas another study reported that approximately 90% of symptoms improved after providing KM treatment interventions for symptoms associated with Sanhupung [8]. Even outside of Korea, it has been reported that approximately 28% of women manage their postpartum symptoms with acupuncture, massage, and exercise as part of an integrated medical approach to assist postpartum recovery [14]. Studies have reported that a combination therapy of scalp acupuncture and electroacupuncture [15], indirect moxibustion therapy [16], and herbal medicine [17] is an effective intervention for postpartum depression.

Although the effectiveness of KM intervention for psychological and physical problems during the postpartum period has been reported, there is a lack of qualitative studies that describe the opinions of mothers [18,19]. A qualitative study is a research method designed to gain a deep understanding of a phenomenon that may be difficult to understand by quantitative analysis, and identifying it within context [20]. Accordingly, our research team conducted a qualitative study on a KM-based postpartum health care program, which is a public health program managed by the National Medical Center (NMC) (Seoul, Korea) since 2017. This study aimed to empathize with Korean mothers by understanding the postpartum experience, and identify experiences, barriers, strengths, and areas for improvement in KM-based postpartum health care programs.

## 2. Materials and Methods

### 2.1. Study Population

The participants in this study were mothers who participated in the KM-based postpartum health care program run by the Department of Korean Medicine at NMC, and those who agreed to participate in the study were recruited. The researchers spoke to 20 participants and 8 agreed to participate in the study. Study participants were recruited until data saturation. The recruitment period for this study was from April 2020 to May 2021. Recruitment for participation in the KM-based postpartum health care program was displayed on a poster, posted inside the NMC and public health center, and announced on the NMC website. This study excluded women who were deemed unfit to participate due to communication impairment caused by serious psychiatric conditions, intellectual disabilities, mood disorders, and other cognitive conditions.

Mothers who were members of households that qualified for Medicaid beneficiaries or had a median income below 80% and were residents of Seoul based on resident registration were enrolled. Mothers who satisfied the above conditions could apply to be enrolled in the program within 3 months of childbirth. Confirmation of eligibility for the program was conducted by doctors and nurses at the National Medical Center in charge of the program. The program covers the entire cost of KM treatment for diseases that occur after childbirth, such as Sanhupung and postpartum depression, with a cap of 300,000 won. There was no cost to participate in the program. Individualized herbal medicine was prescribed by a Korean medicine doctor through differentiation of syndromes. Differentiation of syndromes is a Korean medical evaluation standard, and it is determined based on the patient’s primary complaints, pulse feeling, and abdominal examination. The Korean medicine doctor who prescribed herbal medicine is a qualified specialist with more than 10 years of clinical experience. Herbal medicine was administered twice a day for 20 days. If necessary, the Korean medicine doctor performed acupuncture, electroacupuncture, cupping, and chuna according to the participants’ symptoms.

### 2.2. Ethical Considerations

This research received approval from the Institutional Review Board of NMC on 7 May 2020 (IRB No. H-2004-116-006).

A brief explanation of the research was given orally during the program participation period, and detailed explanations were provided in the written consent form only to those interested in participating in the study (Supplementary File S1). All participants were informed of the study details: the title, background, objectives, inclusion criteria, and methods of the study; expected duration of the study; potential benefits from participating; adverse events, risks, and discomfort the participants may experience; and compensation and costs. After participating in this study, participants were paid a participation fee of 30,000 won. The participants were also informed that their participation was voluntary, that they would not face any negative consequences from nonparticipation, and that they may withdraw anytime during the study. In such cases, their personal information would be permanently destroyed and not used in the study. Participants were informed that all data would be stored in a double-locked cabinet and electronic data would be saved in a password-protected computer with restricted access for the protection of their privacy and confidentiality of the study data, that all information provided by the participants would be anonymized and would not be released to anyone without their consent, and that interview data would be used for research purposes only. The participants provided written consent to participate after receiving this information.

When participating in the Korean medicine-based postpartum health care program, a questionnaire about symptoms is administered and the Edinburgh Postnatal Depression Scale (EPDS), a screening test for postpartum depression, is provided [21]. If participants EPDS scores were above 13, they were connected to the Central Infertility Depression Center at the National Medical Center. In addition, when it was determined that support from an obstetrics and gynecology department or other department was necessary during participation in the program, cooperation with that department was requested.

### 2.3. Selection of Research Methodology

This study used a thematic analysis method described in [22].

### 2.4. Data Collection

Participants were asked questions structured around the interview guidelines (Supplementary File S2) and follow-up questions based on the participants’ responses.

This study used a theoretical sampling method [20]. Mothers who wanted to participate in the postpartum health care program that satisfied the inclusion criteria were included. The researcher conducted the interviews in person or online on a specific date depending on the preference of each participant.

The interviews, which lasted approximately 60–90 min per participant, were conducted in a conference room within NMC or other places easily accessible to the mothers to ensure the participants felt comfortable. All interviews were recorded with prior consent. If the interviews were recorded using a videoconferencing program, the extracted video file was immediately discarded and only the audio file was used. Recorded data were transcribed verbatim by a co-researcher with an understanding of the research content and the transcripts were subsequently reviewed by the researcher who conducted the interviews. Non-verbal expressions were recorded during the interview as field notes, and were considered when coding the transcription record. One interview was conducted per participant, and any insufficiencies identified after the interview were supplemented through a follow-up telephone interview. In-depth interviews were conducted using semi-standardized open-ended questions, which were designed to allow the participants to freely express their thoughts within a loosely defined framework.

Data were collected until the theoretical saturation point was reached and where it was objectively determined that the data collected for analysis were sufficient and no additional data collection was needed. Ultimately, data were collected through one-on-one in-depth interviews with eight participants.

### 2.5. Data Analysis

Data analysis was performed by qualitative thematic analysis, whereas MS Excel (Microsoft, Redmond, WA, USA) was used for data analysis and the transcribed data were used as raw data. Instead of a batch analysis involving the collection of all the data needed for the study simultaneously, theoretical sampling and a constant comparison method were used, which involves repeated data analyses, as they are collected and then a return to the field to collect additional data, if required. Each concept and category were formed using an inductive approach [20]. Interview transcripts and field notes were read line by line, and the contents considered important were extracted to construct a semantic unit, which is a set of words or sentences related to the same central meaning [23]. Subsequently, the derived semantic units were grouped, and each group was labeled with a code. Codes collected in this manner were classified into categories.

A preliminary analysis was performed by the researcher who conducted the interviews. To assure the objectivity of the analysis process and results, transcribed data and analyzed results were shared with a doctor of KM with experience in qualitative research and revised through advisory and discussion processes. The outside researcher did not directly view patient information or case records and only had access to transcribed data and analyzed results with identifiable information that had been codified.

### 2.6. Rigor of Research

#### 2.6.1. Theoretical Sensitivity of Researcher

Theoretical sensitivity refers to the ability of the researcher to understand a theory and give meaning to what is recognized as being important in relevant data [19]. The researcher who performed the main analysis in this study obtained a KM doctor license after graduating from a KM college in 2018. The researcher encountered many mothers wishing to receive KM-based postpartum health care for Sanhupung while working as a resident at the Department of Korean Neuropsychiatry at NMC from 2019 to 2021. To develop the competencies required by a qualitative researcher, the researcher attended >10 workshops on qualitative research, which covered data collection methods, data analysis methods, and research report writing techniques, and the researcher received training on the basic concepts and performance of qualitative research. Moreover, the researcher gained experience in qualitative research approaches, such as analysis of research articles, by conducting a literature review.

#### 2.6.2. Validity and Reliability of the Research

Triangulation was applied to compare the nonverbal expressions, case records, and audio recordings to ensure reliability and validity. Moreover, an independent researcher reviewed coding by the main interviewer with qualitative research experience using a peer examination technique to check the validity. The quality of the research reporting was enhanced by following the consolidated criteria for reporting qualitative research (COREQ) guidelines (Supplementary File S3) [24]. Transcribed and analyzed data were randomly selected from a table of random numbers generated by MS Excel (Microsoft), and then checked.

## 3. Results

### 3.1. General Characteristics of the Participants

The study population consisted of 8 participants with a mean age of 34 years (range 27–41 years). There was one primipara and seven multiparas. Other general characteristics are presented in Table 1. All participants had taken herbal medicine, and all participants except one had received acupuncture during their visit. Depending on their condition, they also received chuna, electroacupuncture, cupping, and moxibustion therapy. Four patients received one outpatient treatment session and four patients received two sessions. None of the mothers qualified for Medicaid beneficiaries; however, they all had a median income below 80%.

As a result of the EPDS test of the participants in this study, none of the participants had an EPDS score above 13. None of the participants suffered from perinatal mood and anxiety disorder, and no one had received any additional psychiatric care. Moreover, none of the participants needed the help of an obstetrician during the period of study.

### 3.2. Creation of Categories

Meaningful statements regarding the “understanding of the experience and awareness of childbirth and postpartum care and experience of participating in KM-based postpartum health care program” were extracted. A total of 447 codes were derived from constant comparisons of these statements. Codes were categorized into 26 subcategories, which were grouped into 8 representative categories. Eight categories were classified into two major themes: “experience and awareness of childbirth and postpartum care” and “experiences of the KM-based postpartum program”.

Detailed quotes of participants are attached as supplementary tables (Supplementary File S4).

### 3.3. Experience and Awareness of Childbirth and Postpartum Care

Experience and awareness of childbirth and postpartum care were analyzed in 4 primary categories and 12 subcategories (Table 2). The four primary categories were as follows: breakdown of the body and mind; impossibility of postpartum care without help; relentless effort for recovery; and body and mind recovery, and the details are as follows.

#### 3.3.1. Category 1: Breakdown of Body and Mind

The participants often faced the situation where they were responsible for both childcare and housework and would meet the needs of the baby without addressing their own needs. They stated that they became exhausted and experienced breakdown of their body and mind and felt that they had lost a lot since childbirth. In the non-verbal expression, categories derived in accordance with the verbal expression were supported. When discussing this category, the faces of the participants were dark, but there were no tears visible. During the interview, they did not appear to require psychiatric help.

##### Subcategory 1: Body Weakened by Childbirth

The participants stated that their body had become weak from childbirth itself and that postpartum symptoms became more severe as parity increased. They stated that they experienced a variety of postpartum symptoms, including pain, chills, swelling, weakness, loss of appetite, and fatigue:

“*It takes a toll on the body, really. I can feel it. I was definitely more energetic after my first childbirth, but with the second and third, my body just kept breaking down more and more.*”

(Participant 1)

##### Subcategory 2: Physical and Mental Exhaustion from Both Childcare and Housework

The participants stated that they repeatedly experienced insomnia due to childcare and became increasingly more physically fatigued while also becoming mentally exhausted because of emotional dysregulation. Those who had other children reported no time to rest due to having to take care of all of the children at once and because they had no choice but to continue with breastfeeding or housework, and their pain would fluctuate in severity. Contrarily, they also expressed worries and concerns about childcare:

“*About 3 weeks after leaving the postpartum care facility, (the baby) wouldn’t sleep at night and kept throwing up after eating, having to stay up at nights continued, which made it physically and mentally difficult. I was thinking ‘did I do something wrong to the baby’…*”

(Participant 4)

##### Subcategory 3: The Hardship That Comes from Having to Deal with It Alone

Participants stated that it was too much to care for the babies all day at home by themselves. In particular, things that the husband could not help with, such as breastfeeding, or things that they were solely responsible for caused stress since no one else could relieve them of these duties. Participants could use breast pumps or stored milk depending on their circumstances, but they thought it was something only mothers could do:

“*If it was formula, a man can handle it, but breastfeeding is something I have to do. My husband goes to work at 7 AM and comes home at 9 PM, but all that time it is just me and the baby… Breastfeed the baby, clean the house, do the laundry, and whenever the baby cries, I have to soothe, hug, and caress the baby. I also have to communicate with the baby, so it’s all up to me.*”

(Participant 3)

##### Subcategory 4: Postpartum Depression Due to a Sense of Loss and Lack of Communication

Participants stated that they experienced depression due to a sense of loss from feelings of social isolation, career interruption, and losing their own space after having a baby. They also mentioned there were frequent episodes of conflict with their husband or in-laws and that they experienced postpartum depression due to not having an outlet to discuss their thoughts or help people understand their situation. Although mothers were able to communicate with each other before, the current COVID-19 pandemic has made this difficult, with people avoiding meetings:

“*And now, we can’t do group activities. So, I have no time to communicate and am all on my own, which makes me depressed. There aren’t many programs… My husband can’t come often… That’s when I became depressed.*”

(Participant 3)

##### Subcategory 5: Difficulty Obtaining Professional Help

Participants stated that even when their body was hurting, it was difficult for them to seek help for various reasons, and that they were barely holding on. Even if they had postpartum depression, counseling was just one session, and it was difficult to connect with the center:

“*I didn’t think (about going to a hospital). Herbal medicine is expensive, and I thought it would get better. Even if I went to a hospital, medication that can be used is limited…*”

(Participant 6)

#### 3.3.2. Category 2: Impossibility of Postpartum Care without Help

Participants mentioned that postpartum care was possible through not only their efforts but also when there was helpful support from the very beginning, such as help from those around them or government financial support. They expressed that postpartum care was easy when they knew who to ask for help and received sufficient help, but it was difficult otherwise.

##### Subcategory 1: Need Help from People around You

Participants expressed that they needed someone to look after the baby when visiting the hospital, and that things were easier when family members helped with housework or childcare; however, they faced difficulties when such help was not available:

“*When I received postpartum care and gave birth, for me, it was my third child. I had to have someone look after the older kids… Their dad was there, but he had to be with his mom, so it was difficult when there was no babysitter. Taking care of the kids… Two of them… That’s somewhat difficult…*”

(Participant 3)

##### Subcategory 2: Financially Burdensome Postpartum Care

Participants expressed difficulties in receiving sufficient postpartum care due to the additional costs involved while already facing an economically difficult situation. They also stated that it was difficult to pay for the cost of herbal medicine for postpartum care as their insurance did not cover it:

“*Can’t consume a lot mostly due to financial issues, postpartum herbal supplements are somewhat expensive to begin with.*”

(Participant 1)

#### 3.3.3. Category 3: Relentless Effort for Recovery

Mothers spared no effort for recovery, including trying to sweat and keep their body warm since they would experience postpartum symptoms when they felt a breeze or it was cold. They also expressed fears about lifelong suffering without proper postpartum care. Therefore, they showed a willingness to take better care of their bodies. They exhibited a strong will for recovery since they believed that they must recover quickly to take care of their children and that mothers must be strong to ensure that things run smoothly at home.

##### Subcategory 1: Awareness of the Path of Recovery from Sanhupung

Based on their previous experience of pain after feeling a cold breeze, mothers believed that they needed to avoid breezes and keep their body warm. They also believed that recovery could be facilitated by sweating, and thus, they deliberately tried to sweat. Some also considered good lochia discharge or blood circulation to be important. They also had herbal medicine prepared in advance based on the belief that herbal medicine could be beneficial for physical recovery:

“*My mother says I shouldn’t let any breeze in at night… I always gave birth around May. May is hot… But, I always wore long sleeves and long pants… I had to sleep with a winter blanket, that way I could sweat for about 2 weeks. She kept asking did I sweat today, since even if it’s hot, I need to sweat for faster recovery.*”

(Participant 1)

##### Subcategory 2: Fear of Sanhupung

Participants expressed concern because they had heard that they would experience pain in many areas without proper postpartum care and that they should rest to recharge their vitality during the postpartum period. They also stated that they were willing to try anything that is beneficial to the body, believing that this may be their last time receiving postpartum care:

“*I worried a lot. It’s my third child and if I don’t take care of my body properly, I may have lifelong chronic disease. This is my last postpartum care, and they say you have to do a really good job on the last postpartum care. Not doing so can cause pain here and there*…”

(Participant 3)

#### 3.3.4. Category 4: Body and Mind Recovery

##### Subcategory 1: Prepare in Advance through Experience

Participants stated that despite the difficulty, once they experienced childbirth and postpartum care once, they were able to prepare in advance as they learned coping methods:

“*With my second child, I had severe postpartum depression… Back then, my joints hurt a lot, but this time, postpartum depression seems less severe than before. Maybe because I have prior experience. I think I’ve gained some know-how about pain during the postpartum care period.*”

(Participant 4)

##### Subcategory 2: Satisfied with Multiple Sources of Support

Some participants mentioned that they were satisfied with financial assistance for postpartum care, such as the KM-based postpartum health care program or various government supports, despite a low income. Due to COVID-19, the government provided relief funds, and there was financial support, such as diaper vouchers and medical support for newborns:

“*For me…my husband has a low income, so I am receiving just about all the support available. So, I’m okay (laugh). My feeling is like, the government does a lot, and this is what I get for all the taxes I’ve paid to date.*”

(Participant 2)

##### Subcategory 3: Depression Relieved through Communication

Participants stated that they relieved depression by actively communicating, such as talking to family members. They also mentioned that it is important to have someone to communicate with:

“*Fortunately, what most mothers talk about and what they find most difficult after giving birth is being unable to communicate. But, for me, my mother lives close by and my sister also lives close by, so I was able to release everything before depression hit. Since I have people who I can communicate with and would come running before I even talked about having difficulties. That made depression go away and I felt a bit relieved even when I became angry and frustrated.*”

(Participant 5)

### 3.4. Experience of KM-Based Postpartum Program

Experiences of the KM-based postpartum program were analyzed based on 4 main categories and 17 subcategories (Table 3). The four main categories were: half doubt at first; effects of managing postpartum symptoms; comprehensive KM management for postpartum women; and suggested improvements for the KM-based postpartum program. The details of the categories are as follows.

#### 3.4.1. Category 1: Participation with Vague Expectations

Participants stated that they participated in the program with the expectation that their body would get better or with ambiguous expectations and skepticism, thinking that it would be better to participate in the program than not. This indicated that they participated thinking that things would get better without knowing specifically what would improve. They also had a vague expectation that all symptoms would improve simultaneously:

“*The reason why I didn’t have any expectations is because everything sponsored by the government is mostly just formality… I came because it’s probably better than nothing. But, it was much better than I thought, so my satisfaction level was really high.*”

(Participant 3)

#### 3.4.2. Category 2: Effects of Managing Postpartum Symptoms

##### Subcategory 1: Discharge the “Bad Stuff” out of the Body

Participants stated that taking herbal medicine affected lochia discharge, such as discharging “bad stuff” from the body or facilitating more lochia discharge:

“*After taking herbal medicine, I had one more lochia discharge, even though it had stopped… Feeling of getting rid of bad stuff or fatigue that lingered in the body?*”

(Participant 3)

##### Subcategory 2: Reducing Pain and Coldness

Mothers stated that the reduction of pain or coldness allowed them to better withstand their daily life. They stated “pain and coldness had improved”, “joints became smoother”, and “was expected about preventing letting in wind and there were actually no coldness symptoms”:

“*The body being cold or cold sensation was improved and joints and areas with pain became, should I say, smoother, well that’s how I felt. Although I only took it for a short period…*”

(Participant 4)

##### Subcategory 3: Warm Body and Reinforced Energy

Participants stated that their body felt warm, and they had a feeling of being protected when they took herbal medicine, with reinforcement of energy. They mentioned that they experienced their body being reinforced with a warm feeling and a recovery of energy:

“*Herbal medicine arrived, so I took it. I got the feeling of being energized. Because my hands and feet became warm, I used to wear layers of clothes, but I feel a warm sensation throughout my body, so I wear short sleeves on many days.*”

(Participant 5)

##### Subcategory 4: Better Appetite and Comfortable Digestion

Participants reported that they regained their appetite and digestion became more comfortable, including soothing digestive discomforts and no stomachaches, and it was more comfortable going to the bathroom:

“*It was good. My insides felt okay, and as I took herbal medicine, I felt my body changing. Going to the bathroom, my stomach hurt several times a day, but there’s no more of that. When I ate, my insides didn’t feel good and if I didn’t eat, then I would get hungry, but no more of that now.*”

(Participant 6)

#### 3.4.3. Category 3: Comprehensive KM Management for Postpartum Women

##### Subcategory 1: My Small Discomfort Is Reflected in the Treatment

Participants stated that they were satisfied with the fact that the herbal medicine preparation was individually tailored and any mention of symptoms, such as the presence of lochia or decreased energy, was immediately addressed in the treatment:

“*I have a severe hacking cough. When my body becomes weak, a hacking cough appears. This time, I had a lot of hacking coughs starting from when I was staying at the postpartum care facility. After coming here and taking herbal medicine, and it was the first thing I mentioned… Now, I don’t have them. The doctor said that ingredients that can strengthen the lungs would be added… This is why I like Korean medicine.*”

(Participant 3)

##### Subcategory 2: Expert’s Meticulous and Kind Care Process

Participants stated that they liked the fact that they could talk comfortably about the discomfort they had since the doctor was trustworthy and kind, enquired about the details of all aspects of their life, and performed meticulous examinations:

“*About the care process, I like that there were many aspects and they asked meticulously about my condition and they were kind. The care process itself often involves the sick patient doing all the talking. The doctor made me feel comfortable so I could talk about my issues one by one in detail. So, I was able to talk about where I was hurting, from where to where. Uh… the doctor even asked whether it was more difficult when providing childcare. The doctor asked about areas that hurt during childcare and discomfort in daily life. The doctor was also very careful about verbal tone or behavior. I felt like I received care with meticulousness and consideration with the doctor able to understand where I was hurting.*”

(Participant 5)

##### Subcategory 3: Safe KM Treatment

Mothers stated that they trusted that the ingredients were good for them and believed that herbal medicine would be less irritating than other types of Western medicine and that herbal medicine was safe enough to take while breastfeeding. On the other hand, even those who distrusted herbal medicine experienced alleviated distrust through the expert care they received:

“*Herbal medicine is good for the body. Especially mothers… Under the same circumstances, it’s better than taking some other strange things… It’s accurate. It’s trustworthy since the doctor is there to accurately take your pulse.*”

(Participant 3)

##### Subcategory 4: Body and Mind Recovered as Well

Participants stated that receiving care for the mind was also good, and understanding and emotional support during consultation. The body also recovered faster than thought:

“*Herbal medicine and acupuncture were satisfactory, but there was a lot of support during consultation. That part actually had a healing effect on me. Right now, I am physically and emotionally sensitive, and to have someone taking an interest and speaking kindly about my childbirth and childcare, that part was good.*”

(Participant 7)

##### Subcategory 5: Continuation of the KM-Based Postpartum Program

Participants stated that the program was practical, and the procedures were simple, and that others should try it and that they should continue to participate. They also mentioned that it would be good to continue to receive regular care:

“*Yes, yes, I will definitely continue… I think it’s a good program. I also think it’s a practical program. There are benefits that you can really feel. It’s that type of program. The procedures are not that complicated. The procedures are simple…*”

(Participant 3)

##### Subcategory 6: Time to Focus on Only Me

Participants stated that the care focusing on mothers, not babies, was satisfactory and they liked the fact that the time spent on care was solely for themselves. They also mentioned that their experience involved learning something new about their own body and mind as they focused on themselves:

“*Receiving treatment and taking medicine, feels like I’m being cared for? Paying attention not only to the baby but also paying attention to me, I think that led to psychological and physical improvement. I receive care since there was a program for taking care of me. When I go out, everything was about the baby, but now, I think about my own body and taking care, so I think it had a positive influence.*”

(Participant 4)

#### 3.4.4. Category 4: Suggested Improvements for the KM-Based Postpartum Health Care Program

##### Subcategory 1: Requires Continuous Treatment

Participants stated that they felt better after treatment, but the conditions became worse again due to housework and other reasons. Since they participated in only a single session, they wondered whether they should continue to receive treatment:

“*I started hurting again as I cared for the baby. It would be great if I can receive it regularly, but the distance and making the time would be difficult…*”

(Participant 4)

##### Subcategory 2: Difficult to Visit If There Is No Place to Leave the Baby

Participants stated that it was difficult to visit because of the baby and that they need someone with whom to leave their baby. Even when they were receiving treatment, their mind was not at peace, and it made it difficult to receive consistent treatment:

“*The doctor said that coming just once will not be enough so come back if I can. It was good after the treatment, but because I have to keep breastfeeding, it is inevitable that I get lumps. Since my husband is out of town, it is not easy to leave the baby with someone else for me to go to the hospital to receive treatment… It’s satisfactory, but it’s somewhat difficult, receiving treatment…*”

(Participant 7)

##### Subcategory 3: Lack of Awareness and Publicity about KM-Based Postpartum Care

Participants stated that they had not known about KM-based postpartum care until now and that this was the first time they had tried herbal medicine for postpartum care. This indicates a lack of publicity and the need for active promotion for participation in the program:

“*It’s too bad that it has not received enough publicity. Promotion through the Internet or something… There, they didn’t have anything like this… There was no information about this… I think it would be good to promote it through hospitals, gynecologist’s office, or postpartum care facilities.*”

(Participant 6)

##### Subcategory 4: Necessity of Home-Based Treatment and Video-Based Treatment

Participants stated that because they faced difficulties in participating in the program, it would be great to have a care service that came to their home to check their physical condition, and video consultations for additional checking of their physical and mental state. They suggested that video-based care alone could help comfort emotionally depressed mothers:

“*If they are able to provide care as home-visit service…that would be better. Mothers could be more comfortable since the baby would be right next to them while receiving care or talking to the doctor.*”

(Participant 5)

“*Time is like gold to mothers who have to take care of their baby, so they are not able to go back and forth to the hospital. It would be good to do consultations by Zoom or send additional herbal medicine. If not, check where else hurts through Zoom… It would be good to check the complexion and all. That’s because mothers are in a rush…because they want more treatment. When they are alone just with the baby, they also have the feeling that the depressed feeling may go away by talking to another adult. Wouldn’t mothers with a lot of psychological problems be consoled a lot by just having such conversations?*”

(Participant 8)

## 4. Discussion

In this study, in-depth interviews were conducted to identify and analyze the experiences of mothers who participated in a KM-based postpartum care program. This study also identified the comprehensive postpartum care experiences of mothers to determine what problems the mothers faced, what practical needs they have, and how the experience of participating in KM-based postpartum care influenced them. In a previous study, the research team of this study analyzed the trends in research on health care for mothers during the postpartum period. The results showed that there were only five previous qualitative studies, and none of these studies analyzed the efficacy of postpartum care interventions or identified the experiences of mothers, which clearly indicated the need for more qualitative research on this topic [18].

KM-based postpartum care aims to manage Sanhupung by means of Korean medicine interventions, such as acupuncture and herbal medicine. Korean mothers recognize KM-based postpartum care as one of the options, and in a previous study, 43.4% of mothers with Sanhupung were given KM-based postpartum care [25]. One study did quantify the level of satisfaction in using KM-based postpartum care among mothers using a Likert scale, and 85.24% said they were satisfied compared to when they did not receive KM-based postpartum care [13]. However, because the reason for satisfaction was not investigated, it was limited in terms of the identification and understanding of the concept of satisfaction with KM-based postpartum care. Accordingly, it was determined that a study using a qualitative approach that listens directly to the voices of mothers and provides a rich description of the actual challenges in postpartum care and experiences of KM-based intervention programs would be more appropriate.

Most mothers interviewed had experienced breakdown of the body and mind for various reasons after childbirth. With the body already weakened by childbirth, they reported feeling fatigued, exhausted, and depressed from the responsibilities of childcare and housework all on their own. When they did not have anyone to provide emotional support, such as their husband, or experienced family conflicts, they mentioned that it could lead to postpartum depression. Although none of the participants were diagnosed with postpartum depression, they did identify the need to remain vigilant regarding postpartum depression in postpartum care and to pay more attention during the absence of emotional support. The mothers felt a sense of loss with childbirth itself in terms of not having their own space or interruption of their careers, and as a result, they felt that they were the only ones making a sacrifice. Similarly, Park et al. (2022) found that mothers lack adequate free time and being a housewife is a factor that increases fatigue [26]. Moreover, a study of the factors influencing maternal postpartum function in the United States identified emotional regulation, insufficient time for task demands, and engagement with social support [27]. This confirms that postpartum mothers may commonly experience similar difficulties.

Despite this, they were aware that they needed postpartum care and showed relentless effort regarding recovery. Such findings were consistent with results from existing studies indicating that mothers in East Asian countries are aware of the importance of postpartum care [25,28,29]. They not only showed efforts at a personal level, such as keeping the body warm and sweating to prevent Sanhupung, but also requested help from postpartum helpers and family members with childcare and housework to ensure enough rest. However, such requests for help were only possible if they had someone around them who could help; if they did not have anyone, this was not possible. Consequently, it can be inferred that the quality of postpartum care would vary significantly depending on the home environment of each individual.

The uniqueness of Korean postpartum care was also revealed. Korean mothers’ perception of proper postpartum care is that there are six ways to warm their bodies and avoid cold, get some rest, eat well, not overdo it, maintain cleanliness, and take good care of themselves [30]. There is a perception that if this is not done well, problems can develop, such as Sanhupung [31]. According to these cultural characteristics, there is a postpartum care center in Korea [32]. The postpartum care center provides education as to how to care for newborns and breastfeeding, and provides services, such as massages [32]. In total, 81.2% of Korean mothers who gave birth in 2020 received an average of 2 weeks of care at postpartum care centers following childbirth [33]. After this, Korean mothers often received traditional postpartum care at home, and reported that the ideal period of postpartum care was an average of 71 days [33]. According to data from Statistics Korea, over 21 years, postpartum care center users spent an average of 24.31 million won and 815,000 won at home for postpartum care [33]. The cost of postpartum care is not covered by insurance in Korea, so there is a perception that postpartum care should be done well, but the burden is also high. Because of the economic burden, it was not always possible to stay at a postpartum care center or hire a postpartum helper. They also found it difficult to personally pay for herbal medicine, even though they knew it would help their recovery. On the other hand, they also expressed that they had access to good postpartum care due to government support and they were satisfied with the financial aid or services offered by the government. Mothers who were admitted to the program experienced physical and mental recovery through their own efforts and various assistance, including support from the KM-based postpartum care program. They expressed their satisfaction as they recognized faster recovery and gradual improvement.

Similar discussions were had surrounding postpartum experiences in previous qualitative studies [18], but the content of mothers’ experiences with Korean medicine interventions was newly discovered. When the mothers were asked directly about the reasons for participating in the program, their responses indicated that many had applied for the program with skepticism and vague expectations, which could be attributed to the lack of clear awareness about KM-based care. However, mothers were aware that herbal medicine or KM treatment could be helpful in postpartum care, indicating that they believed that KM-based care could be beneficial; however, they did not have a clear understanding. Moreover, it was determined that their vague expectations turned into clear awareness and expectations after receiving actual postpartum care. Inaccurate knowledge spread through word of mouth became accurate knowledge acquired through treatment provided by KM doctors, and the ability of mothers to predict their prognosis made it possible for self-care and increased self-efficacy. Previous studies have reported that 72.11% of Korean mothers had a perception of Sanhupung but that mothers did not have proper knowledge of Sanhupung and did not have a clear understanding of postpartum care methods [25,34]. The findings in this study indicate that there is an opportunity for mothers to access accurate information through KM-based health care.

As the mothers experienced the KM-based health care program, they realized that their level of satisfaction increased as they realized that someone was listening to what they had to say and that they were able to explain their discomfort while they also assessed the program positively in terms of experiencing actual physical recovery. Participants reported that the herbal medicine helped reduce pain, warm the body, and improve digestion. Previous studies have reported that it relieves wrist pain, back pain, pelvic pain, and coccyx pain after childbirth [35,36,37,38]. Meanwhile, cold breeze is a Korean cultural expression. In Korea, it is considered that illness occurs due to exposure to cold wind after childbirth. This idea can be similarly seen in the importance placed on warming the body after childbirth in other Asian countries [39,40,41]. In the sense that herbal medicine warms the body, KM management directly helped with relieving the physical discomfort experienced by Korean mothers while matching their concepts of postpartum care. In addition, satisfaction with pain reduction and digestion improvement were not covered in detail in the usual postpartum care. It could be considered that the KM-based postpartum care pays more attention to pain reduction, body warming, reinforced energy, and improvement of digestion than other routine management of mothers. Korean mothers were concerned regarding this discomfort, believing that it would become chronic if left unaddressed. In this respect, it is necessary for the clinician to identify the mother’s concerns and suggest treatment if necessary.

Participants also believed that herbal medicine can be taken during breastfeeding and that it is safer than using Western medicine during the postpartum period. With respect to herbal medicine, there have been studies on the concentration of heavy metals, residual pesticides, and sulfur dioxide before and after decoction [42] and the effects of Saenghwa-tang on breastfeeding Korean mothers [43,44]. These studies reported no adverse events and no detection of harmful components, which confirmed that taking herbal medicines prescribed by a professional is safe, even during breastfeeding. The use of herbal medicines during pregnancy and postpartum is seen in many countries; however, there are concerns over its improper use [45,46,47,48,49,50,51,52]. Therefore, it may be worth considering the use of herbal medicines prescribed by a specialist for treatment of postpartum symptoms.

Meanwhile, the limitations of KM-based postpartum care reported by the participants included the following: (1) the fact that it was difficult to visit when there was no one with whom to leave the baby, (2) lack of publicity about the program, and (3) the program was a one-time-only program. They also emphasized the need for continuous treatment since it was difficult for them to achieve complete recovery from pain that appears when doing physical work, such as childcare or housework.

With respect to future improvements, they mentioned that it would be good to expand the budget and provide education about postpartum care methods that mothers can enact on their own while also emphasizing the need for a visiting care service for mothers with infants who have difficulty traveling to the program. Moreover, they also mentioned that video consultation could be helpful for relieving depression.

Mothers stated that they felt more depressed from experiencing a sense of isolation with no outlet for emotional exchange and support due to restrictions on gatherings with other mothers and visits to health centers due to the COVID-19 pandemic. Mothers suggested that video consultation could be helpful. Therefore, it appears there is a need for new approaches, including the establishment of a program that includes video interviews with KM doctors for managing postpartum depression during times, such as the COVID-19 pandemic. Futterman et al. (2021) also found that telemedicine was useful for continuing satisfactory prenatal care [53]. Therefore, telemedicine may be useful for pregnant women and postpartum management.

In terms of the opinions of mothers regarding KM treatment, these can be explained as follows.

First, it is not easy to visit a health care institution to receive KM treatment during the postpartum period. Many mothers reported that they did not have people with whom they could leave their child while they received KM-based intervention during the postpartum period. Moreover, although they recognized the need for continuous treatment, it was difficult to make the time for this due to childcare and housework responsibilities. Mothers reported a great need for video consultations or home visits. Future health care policies should include these services.

Second, KM treatment is helpful and appropriate for the management of Sanhupung symptoms and postpartum care. Mothers felt that the discharge of the “bad stuff”, such as lochia; keeping the body warm by avoiding cold breezes that can cause soreness and pain; and improving body weakness that occurs due to the loss of nutrients to the baby should be prioritized. Mothers believed that KM treatment can warm the body and provide supplementation, and as a result, they were keen to receive KM treatment if they had the time to participate. They stated that they felt their body getting warmer after KM treatment and Sanhupung was prevented while also experiencing a reduction in pain due to existing postpartum symptoms.

Third, mothers reported that the treatment time with the KM doctor gave them a sense of psychological stability. Consultation with the KM doctor provided mothers with support and comfort and helped them gain a better understanding of their physical and mental state. With the treatment time focused solely on them, mothers took psychological comfort from seeing their smallest discomfort being addressed and directly reflected in the treatment. Mothers felt that their identity was now based solely on motherhood, and they reported a great sense of loss of identity due to not having time for themselves or interruption of their career, and this often resulted in depression. However, they achieved mental recovery because the treatment time represented time when they could focus solely on themselves.

This study had the following limitations. One limitation was that the researcher was a KM doctor who had observed the KM-based postpartum care program for 3 years. Experiential bias may have been introduced in all stages of the study, including during participant recruitment, interviews, data analysis, and theory generation. Moreover, although the researcher did not meet the participants during the treatment process, the participants may have recognized that the person conducting the interview was a KM doctor. Consequently, they may have been reluctant to share their negative experiences of KM treatments. However, to maintain neutrality in the interview, we specifically attempted to enquire about experiences or anecdotes that the mothers felt while participating in the program, irrespective of whether they were positive or negative experiences. Although caution is needed in interpreting the excessively positive effects of KM postpartum care programs, this study is clinically meaningful in that it is important to understand the pain experienced after childbirth and to provide professional management to address this pain. As a second limitation, a preliminary interview was not conducted, which may have impacted the quality of the interviews. The third limitation is that because the study was conducted on Korean mothers, it may not be representative of other population groups. There may be unique aspects to their reported experience that cannot be generalized to mothers of diverse backgrounds. In addition, the number of participants was very small (*n* = 8). Recruitment was challenging as the number of participants in the postpartum program decreased because mothers were reluctant to visit the hospital due to COVID-19. However, this number of participants is the smallest number for which the data reached the theoretical saturation point.

Despite these limitations, this study is significant in that it provides an in-depth understanding of the experiences of mothers who received KM-based postpartum care, which is lacking in previous studies. Based on the findings of this study, qualitative studies with new perspectives should be welcomed, and follow-up studies of KM-based interventions will be meaningful for the further development of postpartum care.

## 5. Conclusions

This qualitative study of mothers’ experiences of a KM-based postpartum care program conducted by NMC found that although mothers reported that it was not easy to travel to a health care institution to receive KM treatment during the postpartum period, they recognized the importance of such treatment for the management of Sanhupung symptoms and postpartum care. Mothers reported benefits of KM treatment in terms of body warming, prevention of Sanhupung, and pain reduction. Moreover, the treatment time with the KM doctor gave them a sense of psychological stability. In addition, this study identified the need for home- and video-based KM postpartum care programs; these treatment delivery options should be considered in the establishment of future policies.

## Figures and Tables

**Table 1:** General characteristics of the participants.

Participant Number	Age	Delivery Experience	Mode of Delivery	Feeding	Previous Psychiatric Conditions
1	34	Multipara	Natural	Breastfeeding	No psychiatric history
2	34	Primipara	Cesarean	Bottle feeding	No psychiatric history
3	41	Multipara	Natural	Breastfeeding	No psychiatric history
4	32	Multipara	Natural	Mixed feeding	No psychiatric history
5	38	Multipara	Natural	Breastfeeding	No psychiatric history
6	28	Multipara	Natural	Mixed feeding	No psychiatric history
7	41	Multipara	Natural	Mixed feeding	No psychiatric history
8	33	Multipara	Natural	Mixed feeding	No psychiatric history

**Table 2:** Analysis of participants’ experience and awareness of childbirth and postpartum care.

Categories	Subcategories
Breakdown of the body and mind	Body weakened by childbirth Physical and mental exhaustion from both childcare and housework The hardship that comes from having to deal with it alone Postpartum depression due to sense of loss and lack of communication Difficulty getting professional help
Impossibility of postpartum care without help	Need help from people around you Financially burdensome postpartum care
Relentless effort for recovery	Awareness of the path of recovery from Sanhupung Fear of Sanhupung
Body and mind recovery	Prepare in advance through experience Satisfied with multiple sources of support Depression relieved through communication

**Table 3:** Analysis of participants’ experience of a Korean medicine-based postpartum program.

Categories	Subcategories
Participation with vague expectations	
Effects of managing postpartum symptoms	Discharge the “bad stuff” out of the body Reducing pain and coldness Warm body and reinforced energy Better appetite and comfortable digestion
Comprehensive Korean medicine management for postpartum women	My small discomfort is reflected in the treatment Expert’s meticulous and kind care process Safe Korean Medicine treatment Body and mind recovered as well Continuation of Korean Medicine-Based Postpartum Program Time to focus on only me
Suggested improvements for the Korean medicine-based postpartum health care program	Requires continuous treatment Difficult to visit if there is no place to leave the baby Lack of awareness and publicity about Korean medicine-based postpartum care Necessity of home-based treatment and video-based treatment

## Data Availability

All databases are available from the corresponding author upon request.

## Supplementary Materials

The following supporting information can be downloaded at: https://www.mdpi.com/article/10.3390/ijerph19095332/s1, File S1: Informed Consent. File S2: Semi-structured open-ended interview guide. File S3: COREQ (COnsolidated criteria for REporting Qualitative research) Checklist. File S4: Supplementary table about participants quotes.
