# Supplementary material for: Mothers’ Experiences of Childbirth and Perspectives on Korean Medicine-Based Postpartum Care in Korea: A Qualitative Study
Source: Int J Environ Res Public Health. 2022 Apr 27;19(9):5332. doi: 10.3390/ijerph19095332 (PMC9105879; doi:10.3390/ijerph19095332)
Supplement: Supplementary file 1 [file ijerph-19-05332-s001.zip › File S1.pdf]

File S1. Informed Consent ver.1.2

(explanatory text)

TITLE OF STUDY

Research on Evaluation of Postpartum health care program in Korean Medicine: Using Qualitative study on patients' experiences

This study is a qualitative study through the interview method on the Korean medicine postpartum health care program. You should read the instructions and consent form carefully before deciding whether to participate in this study or not. It is important that you understand why this research is being done and what it does. Researcher Doeun Lee, who conducts this study, will explain to you about this study. This study will be conducted only for those who have voluntarily expressed their intention to participate. Please read the following carefully before deciding to participate, and if necessary, discuss it with your family and friends. If you have any questions, your researcher will explain in detail.

Your signature means that you have been told about the study and the risks, and your signature on this document means that you (or your legal representative) wish to participate in this study.

1. PURPOSE OF STUDY

This study is a qualitative study through an interview method on the strengths, obstacles, and activation methods of the Korean medicine postpartum health care program. Based on your opinions, we will present the basis for future related studies or suggest policy directions.

2. RECRUITMENT (Who can Participate in the study)

[Inclusion Criteria]

1. Prior participation in the National Medical Center's Korean Medicine Postnatal Health care Program
2. Voluntary consent to participate in the study

[Exclusion Criteria]

1. Severe mental health status, intellectual disability, mood disorder, and other cognitive problems that impair communication and thus hinder the individual's participation in the interview.

The interview will be conducted among 10 or fewer participants.

3. STUDY PROCEDURES

<u>If you indicate your intention to participate, you can choose one of the following two courses. Depending on your preference, you may agree to participate in the study immediately and (1) conduct an face-to-face interview, or (2) conduct an online interview at a separate date and time.</u>

(1) Face-to-face interview

You will have a conversation with the researcher for 60-120 minutes in a convenient location. You do not need to prepare anything, just tell the researcher how you feel about the question honestly. With your consent, the researcher may take notes or record during the interview.

(2) Online interview

You will have a conversation with the researcher for 60-120 minutes through video conferencing programs (Ex. Zoom, Google Meeting, etc). You do not need to prepare anything, just tell the researcher how you feel about the question honestly. With your consent, the researcher may take notes during the interview or record video and audio of the video conference. Even if video and audio are recorded, video files are immediately discarded and only recorded files are collected.

4. PERIOD

You will be asked to participate in at least one interview (maximum of two) for this study and each interview session lasting for 60-120 min.

5. RISKS

If the interview time is longer than 1 hour, you may be tired from physical and mental fatigue. So, the interview will be held for 1 hour, followed by a 10-min break, and then resumed. If you have any questions about any side effects or risk factors that may arise during your participation in the study, please contact your researcher immediately.

6. BENEFITS

You will receive 30,000 won as a participation fee after participating in this study. We hope that the information obtained from this study may be helpful in identifying experiences, barriers, strengths, and areas for improvement in Korean medicine based postpartum health care programs.

7. CONFIDENTIALITY

Personal information collected from you through your participation in this study is your name, age, account number and contact information, a recorded file of the in-depth interview (<u>video files created in the video conferencing program will be destroyed immediately</u>), transcription, and original signed informed consent. This information is used for research only during the research period, and the collected information is appropriately managed in accordance with the Personal Information Protection Act. Collated information is kept on the researcher's personal computer with a lock and is accessible only to the researcher. We will do

our best to ensure the confidentiality of all personal information obtained through research. Your name and other personal information will not be used when the personal information obtained from this research is disclosed in academic journals or conferences. However, if required by law, your personal information may be provided. In addition, monitor agents, inspectors, and Institutional Review Board can directly view the research results to verify the reliability of the procedures and data of this research within the scope stipulated by the relevant regulations without infringing on the confidentiality of research participants. By signing this consent form, you will be deemed to have known and consented to these matters.

After the completion of the research, in accordance with Article 15 of the Enforcement Rule of the 「Bioethics and Safety Act」, research-related data will be kept for 3 years after the completion of the research. At the end of the retention period, it will be permanently deleted from researcher's personal computer and destroyed.

8. VOLUNTARY PARTICIPATION

You have the right not to participate in this study, and there will be no disadvantage to you if you do not participate in this study. In addition, you may quit at any time even after participating in the study. If you wish to stop participating in the study, please notify the researcher immediately. Upon cessation of participation, your data and personal information will no longer be used for research and will be destroyed by shredding or incineration.

9. CONTACT INFORMATION

If you have any questions about this study or if you have any problems during the study, please feel free to contact the following research staff:

Name: Joohee Seo Tel: 02-2260-7467

If at any time you have questions about your rights as a research participant, please contact the National Medical Center Institutional Review Board at:

National Medical Center Institutional Review Board Tel: 02-2260-7014

Informed Consent ver.1.2

Research on Evaluation of Postpartum health care program in Korean Medicine: Using Qualitative study on patients' experiences

1. I read the description of this study and discussed it with the researcher in charge.
2. I have heard about the risks and benefits and have received satisfactory answers to my questions.
3. I consent to the recording of the interview between me and the researcher in order to conduct the research.
4. I voluntarily consent to participate in this study.
5. I agree to the collection and use of information about myself obtained through this research within the scope permitted by current laws and institutional review board regulations.
6. I consent to direct access to my personal information when the researcher in charge or an authorized representative conducts research or manages the results, and when research institutes, research fund support organizations, and institutional review board conduct factual survey.
7. I may withdraw from this study at any time and I understand that this decision will not harm me in any way.
8. My signature indicates that I have received a copy of this consent form and I will keep a copy until the end of my participation in the study.

Participant	Name:	Signature:	Date:
Legal representative<br>(If necessary)	Name:<br>Relationship with participant:	Signature:	Date:
Witness<br>(If necessary)	Name:	Signature:	Date:

person conducting informed consent discussion	Name:	Signature:	Date:
