# Supplementary material for: Mothers’ Experiences of Childbirth and Perspectives on Korean Medicine-Based Postpartum Care in Korea: A Qualitative Study
Source: Int J Environ Res Public Health. 2022 Apr 27;19(9):5332. doi: 10.3390/ijerph19095332 (PMC9105879; doi:10.3390/ijerph19095332)
Supplement: Supplementary file 1 [file ijerph-19-05332-s001.zip › File S2.pdf]

File S2. Semi-structured open-ended interview guide.

**Understanding of delivery and postpartum care experiences**	
Q1	How have your mind and body changed after the delivery?
Q2	What type of assistance was most needed during your postpartum care?
Q3	What types of financial, social, and medical help would you like?
Q4	How did you care for your body after the delivery? What did you experience during the process?
Q5	What are the unmet needs for postpartum care you have experienced so far?
**In-depth understanding of KM-based postpartum health care experiences**	
Q6	How did you participate in the KM-based postpartum health care program?
Q7	What expectations did you have of the KM-based postpartum health care program?
Q8	What physical and psychological changes did you experience after participating in the KM-
Q9	What were some shortcomings of the KM-<br>based postpartum health care program? How do you want them to be changed?
