# Supplementary material for: Mothers’ Experiences of Childbirth and Perspectives on Korean Medicine-Based Postpartum Care in Korea: A Qualitative Study
Source: Int J Environ Res Public Health. 2022 Apr 27;19(9):5332. doi: 10.3390/ijerph19095332 (PMC9105879; doi:10.3390/ijerph19095332)
Supplement: Supplementary file 1 [file ijerph-19-05332-s001.zip › File S3.pdf]

File S3. COREQ (COnsolidated criteria for REporting Qualitative research) Checklist

Topic	Item No.	Guide Questions/Description	Reported on Page No.
Domain 1: Research team and reflexivity			
Personal characteristics			
Interviewer/facilitator	1	Which author/s conducted the interview or focus group?	N/A
Credentials	2	What were the researcher's credentials? E.g., PhD, MD	Page 4
Occupation	3	What was their occupation at the time of the study?	Page 4
Gender	4	Was the researcher male or female?	N/A
Experience and training	5	What experience or training did the researcher have?	Page 4-5
Relationship with participants			
Relationship established	6	Was a relationship established prior to study commencement?	N/A
Participant knowledge of the interviewer	7	What did the participants know about the researcher? E.g., personal goals, reasons for doing the research	Page 3
Interviewer characteristics	8	What characteristics were reported about the interviewer/facilitator? E.g., bias, assumptions, reasons and interests in the research topic	Page 17
Domain 2: Study design			

Theoretical framework			
Methodological orientation and theory	9	What methodological orientation was stated to underpin the study? E.g., grounded theory, discourse analysis, ethnography, phenomenology, content analysis	Page 3
Participant selection			
Sampling	10	How were participants selected? E.g., purposive, convenience, consecutive, snowball	Page 4
Method of approach	11	How were participants approached? E.g., face-to-face, telephone, mail, email	Page 4
Sample size	12	How many participants were in the study?	Page 4
Non-participation	13	How many people refused to participate or dropped out? Reasons?	Page 2
Setting			
Setting of data collection	14	Where was the data collected? E.g., home, clinic, workplace	Page 4
Presence of non-participants	15	Was anyone else present besides the participants and researchers?	N/A
Description of sample	16	What are the important characteristics of the sample? E.g., demographic data, date	Page 5

Data collection			
Interview guide	17	Were questions, prompts, guides provided by the authors? Was it pilot tested?	N/A
Repeat interviews	18	Were repeat interviews carried out? If yes, how many?	N/A
Audio/visual recording	19	Did the research use audio or visual recording to collect the data?	Page 4
Field notes	20	Were field notes made during and/or after the interview or focus group?	Page 4
Duration	21	What was the duration of the interviews or focus group?	N/A
Data saturation	22	Was data saturation discussed?	Page 4
Transcripts returned	23	Were transcripts returned to participants for comment and/or correction?	Page 5
Domain 3: Analysis and findings			
Data analysis			
Number of data coders	24	How many data coders coded the data?	Page 4
Description of the coding tree	25	Did authors provide a description of the coding tree?	N/A
Derivation of themes	26	Were themes identified in advance or derived from the data?	Page 5
Software	27	What software, if applicable, was used to manage the data?	Page 5

Participant checking	28	Did participants provide feedback on the findings?	Page 5
Reporting			
Quotations presented	29	Were participant quotations presented to illustrate the themes/findings? Was each quotation identified? e.g. participant number	Page 6 to 13<br>File S4.
Data and findings consistent	30	Was there consistency between the data presented and the findings?	Page 6 to 13
Clarity of major themes	31	Were major themes clearly presented in the findings?	Page 5 to 13
Clarity of minor themes	32	Is there a description of diverse cases or discussion of minor themes?	Page 5 to 13
