# Supplementary material for: Mothers’ Experiences of Childbirth and Perspectives on Korean Medicine-Based Postpartum Care in Korea: A Qualitative Study
Source: Int J Environ Res Public Health. 2022 Apr 27;19(9):5332. doi: 10.3390/ijerph19095332 (PMC9105879; doi:10.3390/ijerph19095332)
Supplement: Supplementary file 1 [file ijerph-19-05332-s001.zip › File S4.pdf]

File S4. Supplementary table about participants quotes

Major theme 1: Experience and awareness of childbirth and postpartum care

Subcategory	Participants quotes
**Category 1: Breakdown of body and mind**	
· Body weakened by childbirth	*"It takes a toll on the body, really. I can feel it. I was definitely more energetic after my first childbirth, but with the second and third, my body just kept breaking down more and more." (Participant 1)*<br>*"I was very depressed when I was so bloated during my stay at the postpartum care facility. I had the hardest time, I was bloated, had milk fever, my breasts became swollen and hurt before breastfeeding, and my perineum had not recovered. For about a week after giving birth… Maybe not up to a full week, but the fifth day was the toughest and I think I was most depressed at that time. My body didn't feel good, so…" (Participant 3)*<br>*"My body became very swollen after giving birth. This was my third time, but I became more swollen than the first or second time. I think that's why it took longer for the swelling to go down." (Participant 7)*<br>*"I really didn't regain my appetite this time, when I was at the postpartum care facility or when I got back home. Felt like simply eating because I had to." (Participant 4)*
· Physical and mental exhaustion from both childcare and housework	*"About 3 weeks after leaving the postpartum care facility, (the baby) wouldn't sleep at night and kept throwing up after eating, having to stay up at nights continued, which made it physically and mentally difficult. I was thinking 'did I do something wrong to the baby'…" (Participant 4)*<br>*"The biggest thing is physical exhaustion. I think lack of sleep. Oh, and eating too… At the postpartum care facility, they gave me three meals a day and let me sleep. But now, I'm fully responsible for taking care of the baby, so I take short naps whenever the baby is asleep… They say I am skin and bone because of lack of sleep and not eating enough. I became so skinny with an appearance that was hard to look at." (Participant 5)*<br>*"Actually, I don't really remember by first childbirth, (now) I think I experienced body aches more frequently… I think it's more difficult because I have to take care of my second child while also caring for my first." (Participant 6)*
· The hardship that comes from having to deal with it alone	*"If it was formula, a man can handle it, but breastfeeding is something I have to do. My husband goes to work at 7 AM and comes home at 9 PM, but all that time it is just me and the baby… Breastfeed the baby, clean the house, do the laundry, and whenever the baby cries, I have to soothe, hug, and caress the baby. I also have to communicate with the baby, so it's all up to me." (Participant 3)*<br>*"It's hard taking care of the baby, but it's also hard to take care of myself. I've been living my life taking care of*

	the baby while almost completely neglecting emotional aspects or my aching body…" (Participant 4)
· Postpartum depression due to sense of loss and lack of communication	*"We are raising our children in the master bedroom. I sleep with them in the master bedroom… That's really stressful (mother- and father-in-law in the living room). Because they are continuing to stay, my space used to be the master bedroom and the living room, and since all of that is gone now, I feel stressed." (Participant 2)*<br>*"And now, we can't do group activities. So, I have no time to communicate and am all on my own, which makes me depressed. There aren't many programs… My husband can't come often… That's when I became depressed." (Participant 3)*<br>*"I am resentful of the fact that my husband doesn't understand that it's hard on everybody and I find it difficult to be in this situation, why I have to live this way, just staying home all the time to take care of the baby. I resent my second child for no reason." (Participant 4)*
· Difficulty getting professional help	*"You have to wait a long time for the government-sponsored psychological counseling center and it was difficult to make an appointment." (Participant 4)*<br>*"I didn't think (about going to a hospital). Herbal medicine is expensive, and I thought it would get better. Even if I went to a hospital, medication that can be used is limited…" (Participant 6)*
**Category 2: Impossibility of postpartum care without help**	
· Need help from people around you	*"When I received postpartum care and gave birth, for me, it was my third child. I had to have someone look after the older kids… Their dad was there, but he had to be with his mom, so it was difficult when there was no babysitter. Taking care of the kids… Two of them… That's somewhat difficult…" (Participant 3)*
· Financially burdensome postpartum care	*"Can't consume a lot mostly due to financial issues, postpartum herbal supplements are somewhat expensive to begin with." (Participant 1)*<br>*"We have some financial difficulties, so we live in a one-room home. There are two children, my husband, and me in that one room, so if a helper comes, it becomes too crowded and uncomfortable. But, even though it's difficult, we tried without a helper. Since we were able to do it with our first child, we can face it head on hoping to do well." (Participant 5)*<br>*"I'm the only income earner, so when getting a sitter for two kids, it becomes a burden." (Participant 3)*
**Category3 : Relentless effort for recovery**	
· Awareness of the path of recovery from Sanhupung	*"My mother says I shouldn't let any breeze in at night… I always gave birth around May. May is hot… But, I always wore long sleeves and long pants… I had to sleep with a winter blanket, that way I could sweat for about 2 weeks. She kept asking did I sweat today, since even if it's hot, I need to sweat for faster recovery." (Participant 1)*
· Fear of Sanhupung	*"I worried a lot. It's my third child and if I don't take care of my body properly, I may have lifelong chronic disease. This is my last postpartum care, and they say you have to do a really good job on the last postpartum*

	care. Not doing so can cause pain here and there...” (Participant 3)
**Category4 : Body and mind recovery**	
· Prepare in advance through experience	*“The first one was such a struggle… My body was more important, but the second, third… (laugh) Because I know it’s nothing serious when they cry…” (Participant 1)*<br>*“With my second child, I had severe postpartum depression… Back then, my joints hurt a lot, but this time, postpartum depression seems less severe than before. Maybe because I have prior experience. I think I’ve gained some know-how about pain during the postpartum care period.” (Participant 4)*
· Satisfied with multiple sources of support	*“For me…my husband has a low income, so I am receiving just about all the support available. So, I’m okay (laugh). My feeling is like, the government does a lot, and this is what I get for all the taxes I’ve paid to date.” (Participant 2)*<br>*“Without this (KM-based postpartum health care program), I would actually have to be treated at a hospital since there’s nothing else like this. It’s not like I could leave my child with someone and say I’ll be back, but receiving this was very helpful. Money is always short…” (Participant 6)*
· Depression relieved through communication	*“Fortunately, what most mothers talk about and what they find most difficult after giving birth is being unable to communicate. But, for me, my mother lives close by and my sister also lives close by, so I was able to release everything before depression hit. Since I have people who I can communicate with and would come running before I even talked about having difficulties. That made depression go away and I felt a bit relieved even when I became angry and frustrated.” (Participant 5)*<br>*“Husband… Baby cries a lot and cannot sleep, and besides those things, my anger level goes up when my husband doesn’t participate. I talked about those things…but, I talk about it right away when I still have the feeling of doing it all by myself sometimes. Do it, like this… He accepts it well when it’s like that…” (Participant 6)*

Main theme 2: Experience of KM-based postpartum program

Subcategory	Participants quotes
**Category1: Participation with vague expectations**	
	“I thought that if I eat this, my body would get better.” (Participant 1)<br>*“I had big expectations. Will Sanhupung really get better now? I also thought it will completely disappear…” (Participant 2)*

	"The reason why I didn't have any expectations is because everything sponsored by the government is mostly just formality… I came because it's probably better than nothing. But, it was much better than I thought, so my satisfaction level was really high." (Participant 3)
**Category2: Effects of managing postpartum symptoms**	
· Discharge the "bad stuff" out of the body	*"I think (lochia) came out more in the beginning. After taking herbal medicine, it was discharged more in the beginning, but it stopped after 1 week." (Participant 1)*<br>*"After taking herbal medicine, I had one more lochia discharge, even though it had stopped… Feeling of getting rid of bad stuff or fatigue that lingered in the body?" (Participant 3)*<br>*"I liked the fact that taking herbal medicine resulted in a faster and greater amount of lochia discharge. Lochia is like final fluid, so some leftover stuff was also discharged. More came out… It was good." (Participant 8)*
· Reducing pain and coldness	*"The body being cold or cold sensation was improved and joints and areas with pain became, should I say, smoother, well that's how I felt. Although I only took it for a short period…" (Participant 4)*<br>*"Yes, at first, I was sore and had pain, it was like neuralgia… it hurts even with just a little bit of wind going through. Before then (KM-based program), I had a feeling of soreness with just a slight breeze, so I used to put on a cardigan. Now, there is no such feeling during my daily activities. That slight breeze used to make me sensitive and irritable. But since I don't have that any longer, I can carry on with daily activities more comfortably… My body was shivering, and I had pain on the left side of my body like wind disease symptoms, but I felt okay after acupuncture on that day." (Participant 5)*
· Warm body and reinforced energy	*"Herbal medicine arrived, so I took it. I got the feeling of being energized. Because my hands and feet became warm, I used to wear layers of clothes, but I feel a warm sensation throughout my body, so I wear short sleeves on many days." (Participant 5)*<br>*"My body tends to be cold, so I took some even before giving birth… Every time I took it, my body warmed up and I liked it since I can definitely feel it." (Participant 8)*
· Better appetite and comfortable digestion	*"It was good. My insides felt okay, and as I took herbal medicine, I felt my body changing. Going to the bathroom, my stomach hurt several times a day, but there's no more of that. When I ate, my insides didn't feel good and if I didn't eat, then I would get hungry, but no more of that now." (Participant 6)*<br>*"I told him that I have constipation, not sure if that's due to breastfeeding… The doctor said that helping with going to the bathroom will be taken care of, so I don't know if that's the reason, but going to the bathroom has become more comfortable, although it is not completely better." (Participant 7)*
**Category3: Comprehensive Korean medicine management for postpartum women**	
· My small discomfort is reflected in the treatment	*"I have a severe hacking cough. When my body becomes weak, a hacking cough appears. This time, I had a lot of hacking coughs starting from when I was staying at the postpartum care facility. After coming here and*

	taking herbal medicine, and it was the first thing I mentioned… Now, I don't have them. The doctor said that ingredients that can strengthen the lungs would be added… This is why I like Korean medicine." (Participant 3)<br>*"Today, I mentioned not having any energy, and then the doctor gave me acupuncture specifically for that. It was good." (Participant 6)*
· Expert's meticulous and kind care process	*"About the care process, I like that there were many aspects and they asked meticulously about my condition and they were kind. The care process itself often involves the sick patient doing all the talking. The doctor made me feel comfortable so I could talk about my issues one by one in detail. So, I was able to talk about where I was hurting, from where to where. Uh… the doctor even asked whether it was more difficult when providing childcare. The doctor asked about areas that hurt during childcare and discomfort in daily life. The doctor was also very careful about verbal tone or behavior. I felt like I received care with meticulousness and consideration with the doctor able to understand where I was hurting." (Participant 5)*<br>*"The doctor checked with (his/her) hands like this, more than what I had mentioned. The doctor really took good care, recommending that I should also get other parts treated." (Participant 7)*
· Safe Korean medicine treatment	*"Herbal medicine is good for the body. Especially mothers… Under the same circumstances, it's better than taking some other strange things… It's accurate. It's trustworthy since the doctor is there to accurately take your pulse." (Participant 3)*<br>*"Since herbal medicine is prepared specifically for mothers, it has good ingredients. That's helpful. Now, I can get acupuncture without a major burden or a heavy toll on the body. I like it since there is no burden of major risk with breastfeeding at home… It was less irritating on the body, and I was hesitant about injections since drugs are being injected, but acupuncture actually doesn't do that. I think it would be good to receive regular Korean medicine treatment." (Participant 4)*<br>*"Actually, I don't have a good opinion of herbal medicine… The doctor told me that I can take it without worrying, so even though I had distrust, I was able to relieve my distrust, so I took it." (Participant 7)*<br>*"Seems like there is no burden to take herbal medicine even when breastfeeding… You can't take western medicine. I think that's why people are turning to herbal medicine more and more." (Participant 8)*
· Body and mind recovered as well	*"Since it also included Korean medicine treatment at the end, it felt like it was a good way to finish up. I recovered faster than expected even though I'm older than when I had my first and second child and the third child would put a bigger strain on my body." (Participant 1)*<br>*"It was really good while doing the consultation. After giving birth, I received psychological support and was thankful that the questions were specifically about me, like whether I have any difficulties… It was good that the doctor also played a role in consultation. Emotional support, aspects like that… Treating the body is good, but I believe consultation is also good." (Participant 4)*

	"Herbal medicine and acupuncture were satisfactory, but there was a lot of support during consultation. That part actually had a healing effect on me. Right now, I am physically and emotionally sensitive, and to have someone taking an interest and speaking kindly about my childbirth and childcare, that part was good." (Participant 7)
· Continuation of Korean medicine-based postpartum program	*"Yes, yes, I will definitely continue… I think it's a good program. I also think it's a practical program. There are benefits that you can really feel. It's that type of program. The procedures are not that complicated. The procedures are simple…" (Participant 3)*<br>*"The doctor supported me well as if (he/she) knew from just looking at the questionnaire and said nice things, which was a source of strength. I thought it would be good to participate regularly." (Participant 4)*
· Time to focus on only me	*"Receiving treatment and taking medicine, feels like I'm being cared for? Paying attention not only to the baby but also paying attention to me, I think that led to psychological and physical improvement. I receive care since there was a program for taking care of me. When I go out, everything was about the baby, but now, I think about my own body and taking care, so I think it had a positive influence." (Participant 4)*<br>*"Once I came, I received comprehensive care, so it seemed that I was shortsighted about being just Korean medicine. Actually, instead of going to a regular hospital, regular hospitals rush everything and come back after talking only about specific areas with pain. But, I guess because it's a postpartum care program, they gave a lot of thought to mothers, which was different and good." (Participant 4)*
**Category 4: Suggested improvements for the Korean medicine-based postpartum health care program**	
· Requires continuous treatment	*"I started hurting again as I cared for the baby. It would be great if I can receive it regularly, but the distance and making the time would be difficult…" (Participant 4)*<br>*"Because it ended after a single session, can I make an appointment and go again? I thought about it but couldn't ask, so I couldn't continue with the program… It ended without any mention of continuing. So, at that point, I thought this is how much medicine I'm supposed to take, and no treatment is needed. I felt the rest was up to me, so I didn't go to the hospital and held out just taking Tylenol." (Participant 4)*<br>*"I felt better, probably because my circulation improved after acupuncture. You're not supposed to move around after acupuncture, but I moved around to do housework and hold the baby, so, I'm not sure since it's something I should continue to keep an eye on." (Participant 5)*<br>*"Soreness, I'm still somewhat sore… It didn't disappear completely, but it's gotten a bit better than the day I went in for the treatment… I think it would be helpful if they create a service that offers house calls for acupuncture." (Participant 7)*
· Difficult to visit if there is no place to leave the baby	*"I wanted to come earlier, I think I came around the 60th day? I think that was the case. It took that long to take a breath; can I take my baby? Something like that… Because of the baby, was it Chuna? Something like that would be good, but couldn't do it. Honestly, I wanted to receive acupuncture, but couldn't do it because of the*

	baby. When the KM doctor felt the area, it hurt a lot, but after receiving acupuncture, it didn't hurt at all. That's why I want acupuncture… I can't because of the baby… Then, someone must come with me, but my husband is working so he uses vacation time from work." (Participant 1)<br>*"If someone who can help is nearby, then I can visit. Because people who do exclusive breastfeeding face difficulties. I have to pump the breastmilk and leave the baby with someone because I need to receive treatment…" (Participant 5)*<br>*"The doctor said that coming just once will not be enough so come back if I can. It was good after the treatment, but because I have to keep breastfeeding, it is inevitable that I get lumps. Since my husband is out of town, it is not easy to leave the baby with someone else for me to go to the hospital to receive treatment… It's satisfactory, but it's somewhat difficult, receiving treatment…" (Participant 7)*
· Lack of awareness and publicity about Korean medicine-based postpartum care	*"I think it would be good to offer herbal medicine for postpartum care or other Korean medicine-based care. I heard that women should take herbal medicine for blood stasis throughout their life. But, most people don't know so they don't take it." (Participant 1)*<br>*"It's too bad that it has not received enough publicity. Promotion through the Internet or something… There, they didn't have anything like this… There was no information about this… I think it would be good to promote it through hospitals, gynecologist's office, or postpartum care facilities." (Participant 6)*
· Necessity of home-based treatment and video-based treatment	*"If they are able to provide care as home-visit service…that would be better. Mothers could be more comfortable since the baby would be right next to them while receiving care or talking to the doctor." (Participant 5)*<br>*"When you do postpartum care at home, you sometimes need to go to the hospital. Because you have pain here and there…so, it's like since my body is in pain, it would be great for someone to visit to take a look… I can't go (to the hospital). Because of COVID-19, I just stay home even though I have pain since it's not a good idea to go to the hospital or take your baby with you." (Participant 8)*<br>*"Time is like gold to mothers who have to take care of their baby, so they are not able to go back and forth to the hospital. It would be good to do consultations by Zoom or send additional herbal medicine. If not, check where else hurts through Zoom… It would be good to check the complexion and all. That's because mothers are in a rush…because they want more treatment. When they are alone just with the baby, they also have the feeling that the depressed feeling may go away by talking to another adult. Wouldn't mothers with a lot of psychological problems be consoled a lot by just having such conversations?" (Participant 8)*
